# Impact of resection margin status and revision transoral laser microsurgery in early glottic cancer: analysis of organ preservation and local disease control on a cohort of 153 patients

**DOI:** 10.1016/j.bjorl.2020.09.008

**Published:** 2020-10-17

**Authors:** Carmelo Saraniti, Francesca Montana, Enzo Chianetta, Giuseppe Greco, Barbara Verro

**Affiliations:** Università degli Studi di Palermo, Dipartimento di Biomedicina, Neuroscienze e Diagnostica avanzata (BiND), Sezione di Otorinolaringoiatria, Palermo, Italy

**Keywords:** Laryngeal cancer, Squamous cell carcinoma, Laser surgery

## Abstract

**Introduction:**

Transoral laser microsurgery represents the treatment of choice for early glottic cancer. Its use and effectiveness are mainly related to laryngeal exposure and deep extension of tumor. Histopathologic assessment of surgical margin presents a main issue about transoral laser microsurgery and complete oncological excision.

**Objective:**

The aim was to analyze the impact of revision surgery on organ preservation and local disease control in patients with early glottic cancer treated by transoral laser microsurgery.

**Methods:**

We carried out a retrospective study on a cohort of 153 patients with early glottic cancer (Tis, T1, T2) treated by transoral laser microsurgery. Resection margins were classified as follows: “free” if macroscopic margin-tumor distance was at least 2 mm, as “close” if it was less than 2 mm and “positive” if the margin was involved by carcinoma. Patients were divided into two groups: patients with free resection margins (Group A) and patients with positive, close or not-evaluable resection margins (Group B). Group A (36) underwent periodic followup. Group B (117) underwent a second look laser CO_2_ 2 months after surgery. Fifteen patients of Group A with suspected persistence of carcinoma during followup underwent a second laser resection after a time interval of 4–8 months after first surgery. Overall survival, disease-free survival, disease-specific survival, ultimate local control with laser alone and organ preservation rates were estimated.

**Results:**

Five-year overall survival rate and 5-year disease-specific survival were 100% in both groups. The five-year laryngeal preservation rate was 100% in Group A and 95.2% in Group B. Five-year disease-free survival was 92.15% and 5-year ultimate local control with laser alone in 92.15% of patients.

**Conclusion:**

This study has demonstrated that revision Transoral Laser Microsurgery is able to confirm the oncological radicality in most cases, even in the case of positive, close or non-evaluable margins. Considering our results, according to our experience, the second look with CO_2_ laser is a therapeutic strategy to consider, even in the case of close or non-evaluable as well as positive margins.

## Introduction

To date transoral laser microsurgery (TLM) is considered the treatment of choice for early glottic cancer, compared to other therapeutic strategies, such as open surgery and radiotherapy (RT). Indeed, TLM has several advantages over RT and open surgery, including safe resection of lesion and good local disease control, lower morbidity, shorter time of hospitalization and lower costs.^1^, ^2^, ^3^, ^4^, ^5^ However, the use of TLM and its effectiveness are mainly related to laryngeal exposure and deep extension of tumor. Moreover the histopathologic evaluation of surgical margin status represents a main issue with TLM and radical oncological excision; in fact, shrinking of margins during histologic preparation, thermal damage caused by CO_2_ laser beam and specimen orientation due to small size are the main causes of problematic surgical margin assessment, ensuing different managements in the postoperative period.^2^, ^6^, ^7^, ^8^, ^9^ Furthermore, to date there is not agreement on safe resection margin in order to define them as close and/or positive.^8^, ^9^, ^10^, ^11^, ^12^, ^13^, ^14^, ^15^, ^16^, ^17^, ^18^

The aim of our study is to analyze the impact of revision TLM on organ preservation and local disease control in patients with early glottic cancer (pTis, pT1, pT2) treated by TLM. Finally, we evaluated overall survival (OS), disease-specific survival (DSS), disease-free survival (DFS) and organ preservation (OP).

## Methods

### Patients data

We carried out a retrospective study on a cohort of 153 patients with early glottic cancer (Tis, T1, T2), N0M0, treated by TLM from January 2005 to January 2014 in our Ear, Nose & Throat Unit. This study was approved by the Ethical Committee (approval no. 11/2019) and informed consent was obtained from each patient in accordance with the Helsinki declaration.

Eligible criteria were: (1) Early glottic squamous cell carcinoma (pTis, pT1, pT2), (2) Previously untreated glottic cancer, (3) Proper glottic exposure, (4) Absence of contraindications to general anesthesia, (5) Absence of neck and/or distant metastases (N0M0), (6) No cancer extension to paraglottic space or subglottic space no more than 5 mm, (7) Over 5 years followup.

Exclusion criteria were: (1) Glottic carcinoma other than different from squamous cell carcinoma, (2) Previous RT, chemotherapy and/or head or neck surgery, (3) Other coexistent and/or previous tumors.

Before TLM, glottic lesions were evaluated by video-laryngoscopy with a flexible endoscope. A preoperative neck contrast-enhanced computed tomography (CT) was performed in case of cancer extension to anterior commissure and/or ventricle and/or paraglottic space.

### Surgical technique

Transoral laser microsurgery was performed under general anesthesia in all the patients. CO_2_ laser resection was performed with a power setting of 2–6 Watt (W), in superpulse mode, with a beam spot of 1.3 mm.

Cordectomy was graded according to the European Laryngological Society (ELS) (2007)^19^: Type I, sub-epithelial; Type II, sub-ligamental; Type III, trans-muscolar; Type IV, total; Type V, extended; Type VI, anterior commissurectomy with bilateral anterior cordectomy.

Tumor staging was performed according to TNM classification of the American Joint Committee on Cancer (AJCC) (2017 Edition).

Endoscopic resections were performed using en-bloc techniques whenever it was possible, or piece-meal techniques depending on cancer size and localization and glottic exposure. Surgical specimens were oriented and marked with suture knots and black ink. Resection margins were classified as follows: Free – macroscopic margin-tumor distance at least 2 mm; Close – macroscopic margin-tumor distance less than 2 mm; Positive – margin involved by carcinoma.

Patients were divided into two groups: patients with free resection margins (R0) (Group A) and patients with positive (R2), close (R1) or not-evaluable (Rx) resection margins (Group B). All patients of Group A underwent followup.

### Revision surgery

Group B underwent a second TLM surgery under general anesthesia 2 months after the first surgery.

Group A with a high endoscopic suspicion of persistence of carcinoma during followup underwent a second laser resection after a time interval of 4–8 months after first surgery.

### Follow-up

All patients were endoscopically evaluated every month during the first 6 months, every 2 months during the following 6 months, every 6 months during the second year and once a year for the following 2 years.

Persistence of carcinoma was considered when cancerous lesions were histologically detected within 6 months from the first surgery, whereas recurrence was defined as histological evidence of carcinoma after 6 months or more after the first surgery.

### Statistical analysis

OS, DFS, DSS, ultimate local control with laser alone and OP rates were assessed. In particular DFS was calculated using Kaplan–Meier method.

OS was defined as the time between surgery and last follow-up or death. DFS was defined as the interval between last cordectomy and last follow-up visit or relapse. Ultimate local control with laser alone analyzed patients who were successfully retreated with TLM as far as they need open surgery or RT. OP was considered from TLM surgery to laryngectomy or last endoscopic evaluation.

## Results

Our study included 153 patients: 144 men and 9 women, with a mean age of 64 years (range 39–82 years). Mean follow-up period was 75 months (range 60–156 months).

96 patients underwent type II cordectomy (62.7%), 6 patients type III (3.9%), 18 patients type IV (11.8%), 27 patients type V (17.6%) and 6 patients type VI (3.9%). No type I cordectomy was performed in our sample (Table 1).

**Table 1:** Patients and tumor characteristics at the time of first surgery.

Characteristics	No.	%
Patients	153	100
**Age**		
*Mean*	64	
*Range*	39–82	

**Sex**		
*Male*	144	94.1
*Female*	9	5.9

**Type of cordectomy**		
*I*	0	0
*II*	96	62.7
*III*	6	3.9
*IV*	18	11.8
*V*	27	17.6
*VI*	6	3.9

**pT**		
*pTis*	48	31.4
*pT1a*	48	31.4
*pT1b*	12	7.8
*pT2*	45	29.4

**Margin status**		
*Group A*		
R0	36	23.5
Rx	46	30
*Group B*		
R1	43	28.1
R2	28	18.3

	117	76.5

Tumor staging according to pathological evaluation (pT) was: 48 cases (31.4%) with pTis, 48 cases (31.4%) with pT1a, 12 cases (7.8%) with pT1b, 45 cases (29.4%) with pT2 (Table 1).

Histopathologic evaluation of resection margin after first surgery revealed 36 (23.5%) cases included in Group A and 117 (76.5%) cases in Group B, which included 46 not-evaluable margins (30%), 43 close margins (28.1%) and 28 positive margins (18.3%) (Table 1).

We did not observe any intraoperative or postoperative complications. Hospitalization lasted 1–2 days.

Group B (*n* = 117) underwent a second TLM surgery 2 months after the first surgery. The new histological results were absence of disease or mild to moderate dysplasia in 75 patients (64.1%) and persistence of carcinoma in 42 patients (35.9%) (Table 2). The latter were managed as follows: 33 (78.6%) patients with free resection margins: followup; 9 patients with positive resection margins: 2 (4.8%) open partial horizontal laryngectomy type II, 1 (2.4%) RT, 6 (14.3%) third TLM.

**Table 2:** Management and outcome of patients after second laser resection.

Group	Outcome 1°	Second TLM/Follow-up	Outcome 2°	Third TLM/Follow-up/Other	Outcome 3°	Follow-up/Surgery	LR	TLM/RT	Outcome 4°
A	21	Follow-up							
	15	Second TLM	5 Negative/Dysplasia Mi-Mo	5 Follow-up					
			4 pT1a R0	4 Follow-up					
			2 pT1a R+	2 Third TLM	2 Negative	2 Follow-up			
			4 pT2 R+	4 Third TLM	4 Negative	4 Follow-up	1	RT	

B	117	Second TLM	75 Negative/Dysplasia Mi-Mo	75 Follow-up			4	TLM	3 Tis R0
									1 T1a R0
			12 pTis R0	12 Follow-up					
			15 pT1a R0	15 Follow-up			2	TLM	2 Tis R0
			3 pT1b R+	3 Third TLM	3 Negative	3 Follow-up	3	RT	
			6 pT2 R0	6 Follow-up			2	RT	
			6 pT2 R+	2 OPHL	2 pT2				
				1 RT					
				3 Third TLM	3 pT3 R+	3 Total laryngectomy			

R+, positive resection margins; R0, free resection margins; RT, radiotherapy; TLM, transoral laser microsurgery; Negative, absence of tumour; Dysplasia Mi-Mo, dysplasia mild–moderate; OPHL, open partial horizontal laryngectomy; LR, local recurrences.

Among the latter 6 patients, 3 had a histological diagnosis of absence of carcinoma, whereas 3 were treated with a total laryngectomy due to pT3 diagnosis with positive margins (Table 2).

In Group B, the patients underwent a third laser surgery after an interval of 2–4 months (mean of 3 months) from the previous surgery.

Patients of Group A with high endoscopic suspicious of persistence of carcinoma (*n* = 15) during followup underwent a second laser resection in a period of time between 4 and 8 months after first surgery. The histological assessment after second TLM showed persistence of disease in 10 patients (66.7%) and mild dysplasia or absence of disease in 5 patients (33.3%). Four of these 10 patients with carcinoma underwent followup because resection margins were free, whereas 6 patients underwent a third look due to positive resection margins (Table 2).

During the followup, 12 patients (7.84%) showed local recurrence after a mean period of 9.7 months (8–13 months) from the last TLM. One patient belonged to Group A and the other 11 to Group B. Among these patients, 6 patients underwent a TLM procedure and 6 underwent RT (Table 2).

**Figure 1:**
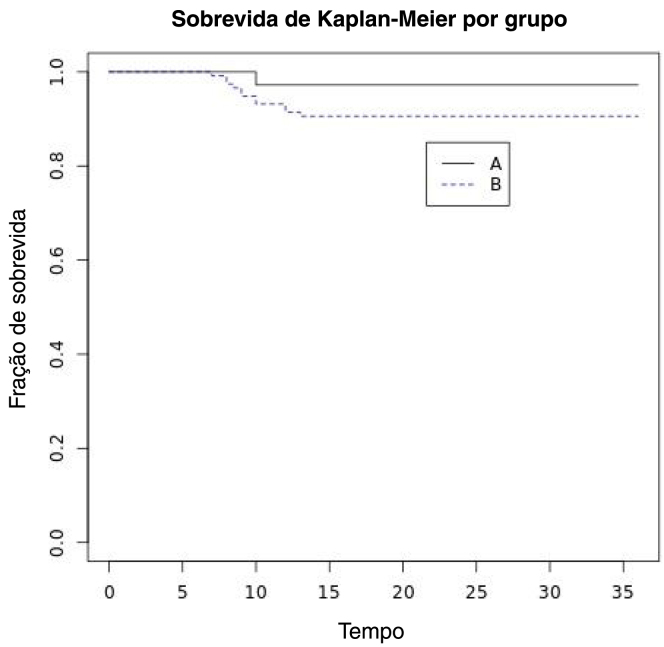
Kaplan–Meier survival curve stratified according to resection margin status.

The 5-year overall survival rate was 100%. Four patients died after more than 5 years due to unrelated causes. Five-year laryngeal preservation rate was 100% in Group A (*n* = 36/36) and 95.2% in Group B (*n* = 112/117). Five-year disease-specific survival was 100% in both groups. Five-year DFS was 92.15% and it was 97.22% in Group A and 90.59% in Group B (Fig. 1). Thus, we achieved 5 year ultimate local control with laser alone in 92.15% (*n* = 141) of patients (Table 3).

**Table 3:** Oncologic outcomes according to margin status.

Oncologic outcome	Total patients		Group A		Group B	
	*n*	%	*n*	%	*n*	%
	153	100	36	100	117	100
5-Years OS	153	100	36	100	117	100
5-Years DFS	141	92.15	35	97.22	106	90.59
5-Years DSS	153	100	36	100	117	100
5-Years OP	112	73.2	36	100	112	95.2
5-Years ultimate local control with laser alone	141	92.15	35	97.22	106	90.59

OS, overall survival; DFS, disease-free survival; DSS, disease-specific survival; OP, organ preservation.

## Discussion

To date TLM represents an effective therapeutic option for early glottic cancers (pTis, pT1 and pT2), resulting in better outcomes than open surgery and radiotherapy in several aspects.^1^, ^3^, ^20^ Indeed, one of TLM advantages is related to its potential to achieve both functional preservation and complete resection of carcinoma, as previously reported.^1^, ^4^, ^6^, ^8^ Hence, over the years TLM techniques have improved up to tailored resections thanks to lesion-customized cordectomies.^21^ Surgical oncological radicality is related to two main factors: laryngeal framework and behavior of carcinoma that may present a superficial and/or deep extension.^1^ Thus, TLM should ensure complete oncological resection and consequently should pay attention to superficial and deep extension towards the paraglottic space and/or anterior commissure. Actually, several authors have reported that anterior commissure involvement could be considered a risk factor for local recurrence^5^, ^7^, ^10^, ^11^ due to insufficient barriers that could prevent extralaryngeal spreading, especially in the case of bad exposure. Moreover, in the literature there is disagreement about margin-tumor distance: Hoffmann and Sigston considered 0.5 mm as safe distance,^8^, ^10^, ^11^ other authors as Wòjcikiewicz, Ansarin, Fiz et al. and others^9^, ^12^, ^13^, ^14^, ^15^ suggested 1 mm as range and Hartl and Charbonnier^16^, ^17^ reported 2 mm.

In our study, we reported a margin status as negative if tumor-margin distance was 2 mm or more. A complete resection of carcinoma depends also on other issues that could make a correct histological assessment of resection margins difficult. These are: (1) shrinkage of the specimen both after resection and after formalin fixation, (2) 0.3 mm area of carbonization all around the excised lesion, (3) CO_2_ laser thermal tissue damage, (4) tumor excision using a piece-meal approach, (5) small size of the specimen that make its orientation difficult and (6) unreliability of frozen sections.^1^, ^3^, ^6^, ^7^, ^17^, ^22^ Moreover, several authors reported the importance of the learning curve on radical excision of tumor and consequently on local recurrence rate.^1^, ^22^ Indeed, in our case series, in order to avoid this bias, all the lesions were treated exclusively by two surgeons with several years of CO_2_ laser experience.

Difficult histological evaluations of resection margins and possible inadequate radical excision of lesions make revision TLM a valid approach in order to guarantee the oncological radicality with the minimum surgical invasiveness, even if in the literature there is disagreement about the management of histological positive or close or not-evaluable resection margins. So, in case of multiple positive superficial margins or positive deep margin, possible treatment policies include a “wait and see” strategy^4^, ^10^ other than a second laser resection^20^, ^21^, ^23^, ^24^ or even two revision TLM regardless of margin status.^25^ However, in 2014 the European Laryngological Society (ELS)^26^ argued that a “second look microlaryngoscopy” is mandatory when margins are positive and recommended in case of close or non-evaluable margins. Actually, several studies reported a correlation between margin status and local recurrence and DFS rates. In particular, Ansarin et al.^12^ and Crespo et al.^27^ reported a higher risk of recurrence and a lower DFS in patients with positive and close margins without further treatment compared to those with negative margins. Moreover, Ansarin demonstrated that when patients with positive or close margins were treated, their DFS was close to those with free margins. On the other side, other authors reported the lack of statistical correlation between resection margin status and recurrence of disease.^6^, ^8^, ^16^, ^28^ Furthermore, del Mundo et al.^29^ demonstrated in their case series that recurrence may occur even in patients with free resection margins. In our study, we performed a second laser resection in patients with positive, close or non-evaluable resection margins and in those with free surgical margins that showed high endoscopic suspicion of persistence of carcinoma during followup. We performed this second surgery 2 months after the first TLM procedure even if the in literature there is not agreement about the timing of second laser resection. Aluffi Valletti^21^ planned a “second look laryngoscopy” within 4–6 weeks, Michel^28^ after 10 weeks; Preuss^25^ suggested the first second look at 8–10 weeks and the second second look at 16–20 weeks.

The minimum time to assure a complete healing of the surgical site is 2 months. Our second laser resection was a revision of the surgical bed of the previous surgery.

In our case series patients were divided into two groups as reported by Michel.^28^ Similar results to our study were reported by other authors.^12^, ^21^, ^28^ Actually, Ansarin^12^ performed a second laser resection in 36 patients with histopathological positive or close margins and 33 of these were proved free from carcinoma.

In our series, the 5-year overall survival rate was 100% in both groups. This data suggested the lack of correlation between OS and resection margin status, as reported by Aluffi Valletti^21^ and Michel^28^ too. The five-year DFS was 92.15%: specifically it was 97.22% in Group A and 90.59% in Group B, demonstrating a local recurrence rate in Group B treated with second laser resection not significantly different from Group A. This finding was also reported by Ansarin^12^ as written above. Indeed, we achieved 5-year ultimate local control with laser alone in 92.15% (*n* = 141) of patients. Similar results were obtained by Peretti with a 5-year ultimate local control with CO_2_ laser alone of 92.7%.^15^ In our study, 3 patients underwent total laryngectomy, 2 OPHL and 7 radiotherapy. Thus, we achieved a 5-year laryngeal preservation rate of 100% in Group A (*n* = 36/36) and of 95.2% in Group B (*n* = 112/117).

However, our study presents some limitations: our retrospective study didn’t include a comparison with a control group, and we did not evaluate the outcome of vocal and swallowing functions. In particular, as regards the first limit, we do not have a control group for ethical reasons.

## Conclusion

This study confirmed the efficacy of TLM in early glottic cancer in terms of oncological radicality and contextual organ preservation and function, primarily due to its reproducibility and low invasiveness. In addition, in our study revision laser surgery has demonstrated to confirm the oncological radicality in a high percentage of patients (86.27%), even in the case of positive, close or non-evaluable resection margins. By contrast, only 7.84% of the total patients included in the study underwent further therapy (RT or open surgery). Therefore, in consideration of the results obtained, according to our experience, the second laser resection is a therapeutic strategy to consider even in the case of close and/or non- evaluable as well as positive margins.

## Funding

This research received no specific grant from any funding agency in the public, commercial or not-for-profit sectors.

## Conflicts of interest

The authors declare no conflicts of interest.
